# Preparation of carbon-supported ruthenium spinel oxide catalyst and application thereof in the oxidation of 5-hydroxymethylfurfural

**DOI:** 10.1098/rsos.240155

**Published:** 2024-08-28

**Authors:** Junchi Zheng, Zhifeng Wang, Qiulan Shi, Lipeng Jiang, Cuiping Yang, Yuan Zhang, Jianbo Zhao

**Affiliations:** ^1^ Engineering Laboratory of Chemical Resources Utilization in South Xinjiang of Xinjiang Production and Construction Corps, Tarim University, Alar, Xinjiang 843300, People’s Republic of China; ^2^ Quality and Technique Supervision Bureau, Alar, Xinjiang, Xinjiang 843300, People’s Republic of China

**Keywords:** preparations, carbon, rutheniums, oxide

## Abstract

Trivalent ruthenium (Ru) can catalyse the oxidation of 5-hydroxymethylfurfural (HMF) to 2,5-furandicarboxylic acid (FDCA). However, the structure of Ru itself is unstable and is prone to aggregation and oxidation, leading to a decrease in catalytic activity. Therefore, it is necessary to prepare a stable, reliable, Ru-based catalyst. Based on the catalytic properties of trivalent Ru, a stable spinel structure with zinc ferrite was designed and loaded onto different carbon supports to prepare a homogeneous and stable Ru-based catalyst. The structure and physico-chemical properties were characterized through scanning electron microscopy, X-ray diffraction, transmission electron microscopy and other techniques, and the catalyst was applied to the oxidation of HMF for the preparation of FDCA. The results show that the prepared magnetic activated carbon-supported Ru-based catalyst has a concentrated particle size distribution in the range of 5–8 nm, with a loading amount of 3.61 at%. It exhibits strong soft magnetism, which is beneficial for Ru loading. Additionally, it can be reused in the oxidation of HMF to prepare FDCA over 10 cycles, with the product yield remaining essentially unchanged. The catalyst prepared in this study is characterized by recyclability and structural stability, making it promising for practical applications.

## 1. Introduction

As early as 2004, 2,5-furandicarboxylic acid (FDCA) was highlighted as one of the most promising bio-based chemicals by the US Department of Energy. Of particular note, FDCA has a conjugated structure similar to that of phthalic acid, making it a potential substitute for the sustainable production of polyamides and polyesters [1]. It has the potential to replace terephthalic acid in future polymer materials [2–5]. Since then, finding commercially viable routes to FDCA has been an on-going subject of research and investment.

Currently, the synthesis pathways for FDCA mainly include 5-hydroxymethylfurfural (HMF) oxidation, hexanedioic acid cyclization and furfural condensation routes [2,6]. Among these, the HMF oxidation route has attracted the most attention [7–9]. However, owing to the presence of hydroxyl, aldehyde and furan ring functional groups in the structure of HMF, its oxidation produces numerous products, including 2,5-furandicarbaldehyde (DFF), 5-hydroxymethylfuroic acid (HMFCA) and 5-formyl-2-furancarboxylic acid (FFCA), making high-efficiency selective oxidation challenging. To achieve efficient oxidation of HMF to FDCA, researchers have extensively studied catalysts, including large-scale homogeneous catalysts (such as Co/Mn/Br) and heterogeneous catalysts (such as platinum, gold, palladium and ruthenium (Ru)-based catalysts) [10–25]. Gorbanev *et al*. conducted experiments with an Ru(OH)x catalyst supported on hydrotalcite and achieved a 95% FDCA yield after 6 h of oxidation at 413 K without alkaline additives [26]. Sohaib Hameed reported the oxidation reaction of HMF on Ru(OH)x/La_2_O_3_ in an alkaline-free system, achieving a 48% FDCA yield [18]. Walt *et al*. first reported the homogeneous Co/Mn/Br/Zr salt-catalysed oxidation of HMF under different reaction conditions for the preparation of FDCA [27]. In summary, although their experimental results achieved decent yields, the stability of the catalysts was compromised owing to factors such as the decomposition of hydrotalcite, catalyst support, Ru leaching and the tendency of Ru nanoparticles to aggregate and oxidize, leading to the deactivation of the catalyst. Therefore, achieving high efficiency and selectivity for Ru-based catalysts in the conversion of HMF to FDCA requires a rational structural design to improve catalyst stability [28]. Research has shown that composite oxides with a spinel structure exhibit excellent thermal stability and chemical compositional flexibility. The structure is represented as XY_2_O_4_ (where X is a divalent metal cation, and Y represents a trivalent metal cation) [29]. Zinc ferrite, as a typical spinel structure metal oxide, also demonstrates soft magnetism, making it an ideal catalyst modification material. However, ZnFe_2_O_4_ nanoparticles tend to aggregate and disperse poorly owing to their high specific surface energy, and ferrates tend to corrode and release metal ions in strong acids (pH = 2–3), which causes the loss of active sites for Ru, leading to a decrease in catalytic performance.

Carbon-supported catalysts possess abundant pore structures, a large specific surface area, easily tunable surface chemical properties, and excellent acid–base resistance. These features increase the contact area between the catalyst and the electrolyte, facilitating the exposure of active sites on the catalyst. Furthermore, carbon-supported catalysts have the advantages of low cost, easy recovery of active components, and favourable conditions for the reduction of metal phases. Additionally, through a review of the literature [30,31], carbon was found to be the optimal support for HMF oxidation reactions. Using carbon as the support not only reduces costs and facilitates the recovery of active components but also provides excellent acid-base resistance, helpful in overcoming the disadvantage of the spinel structure that is prone to the loss of Ru from the active site under strong acidic conditions. Moreover, the resulting products exhibit high stability and recyclability, making them applicable in a wider range of scenarios.

The alkaline co-precipitation method allows Ru to be doped during spinel formation to produce a mixture of spinel and Ru, which is subsequently loaded onto a carbon carrier to produce a stable catalyst system. In order to enhance the magnetic properties of the catalyst, we negatively magnetize the carbon carrier; two modified supports were designed: activated carbon and magnetic activated carbon, both of which are ultimately applied in the oxidation reaction of HMF. This work provides new insights into the preparation and loading of Ru catalysts.

## 2. Experimental

### 2.1. Materials

Zn(NO_3_)_2_·6H_2_O, FeCl_3_·6H_2_O, FeCl_2_·4H_2_O, NaOH and so on were all analytically pure and were sourced from the Sinopharm Group Chemical Reagent Co., Ltd. Ru trichloride and activated carbon (specific surface area of 1800 m^2^ g^−1^) were sourced from Shanghai Aladdin Co., Ltd. A suction filter (SHB-III) was obtained from Gongyi Yuhua Instrument Co., Ltd. The oven (GZX-9246 MBE) was sourced from Shanghai Boxun Industrial Co., Ltd (their medical equipment factory) and an electronic balance (LE204E/02) from METTLER Toledo Instrument Shanghai Co., Ltd.

### 2.2. Synthesis

First, magnetic treatment [32] was conducted on activated carbon to prepare the catalyst support, magnetic activated carbon. Some 5.41 g of hexahydrate ferric chloride and 1.99 g of tetrahydrate ferrous chloride were added to a three-necked flask with 100 ml of distilled water for dissolution. Some 1 g of activated carbon was weighed and added to this three-necked flask. A sodium hydroxide solution was prepared and slowly added to the flask while controlling the pH of the solution to be around 11. The mixture was stirred continuously in an 80°C water bath for 1 h. The resulting magnetic activated carbon was filtered and oven-dried at 60°C for later use.

The preparation of mixtures of Ru-based active components and spinel structures using an alkaline co-precipitation method was then loaded onto the magnetic activated carbon. The precursor solution was prepared by dissolving 0.9288 g of Ru trichloride, 3.2756 g of hexahydrate zinc nitrate and 4.4633 g of hexahydrate ferric chloride in 50 ml of double-distilled water, forming solution A. Some 1 g of magnetic activated carbon was added to solution A, stirred at 40°C for 24 h and then separated by filtration for later use. In another three-necked flask, 12.42 g of sodium hydroxide and 50 ml of double-distilled water were added, and the mixture was stirred until fully dissolved. The soaked magnetic activated carbon was slowly added to the sodium hydroxide solution, and the resulting mixture was reacted at 110°C for 2 h. After cooling to room temperature, the reaction mixture was filtered. The obtained solid powder was washed with a large amount of double-distilled water until neutral and then oven-dried at 80°C overnight. This product was named ZnFe_2_O_4_-Ru/C-Fe, abbreviated herein to Ru/C-Fe.

The preparation method for the activated carbon catalyst and the magnetic activated carbon catalyst was similar, except that no magnetic treatment was applied. The resulting dark brown powder was named ZnFe_2_O_4_-Ru/C, abbreviated herein to Ru/C.

### 2.3. Characterization

Field emission scanning electron microscopy (SEM) was done on a Zeiss Gemini300 thermal field emission SEM; the pore size structure of the catalyst support was observed. Field emission transmission electron microscopy (TEM) was performed on the Tecnai G2 F20 field emission TEM (FEI Co., USA) at a voltage of 200 kV, and the morphology and loading of the catalyst were observed. The energy spectroscopy (TEM-EDS (Energy Dispersive Spectrometer)) attached to the electron microscope was performed on an EDAX GENESIS instrument (USA), and the elemental composition of the catalyst was measured. X-ray diffractometry (XRD) was performed on a Bruker D8 Advance instrument (Bruker, Germany) at a voltage of 40 kV, a current of 40 mA, a step size of 0.02°, using a copper target, and the incoming ray wavelength was 0.15406 nm, so the crystal form and structure of the catalyst were analysed. X-ray photoelectron spectroscopy (XPS) was performed on a Thermo Fisher ESCALAB 250Xi photoelectron spectrometer, Al Kα (*hv* = 1486.6 eV), under an applied power of 150 W, using a 500 μm diameter beam spot; the binding energy was calibrated at C1s 284.8, so the chemical state information of carbon material was studied. The magnetic properties of the sample were measured using a PPMS-9 physical properties tester (QuantunDesig, USA).

### 2.4. Application

To explore the influences of various experimental conditions on the FDCA yield and find the optimal conditions for catalysing the FDCA reaction, variables were controlled. Initial experimental conditions included: 0.5 mmol of HMF, 50 mg of catalyst, 3 ml of dimethyl sulfoxide (DMSO) as the solvent, a reaction temperature of 110°C, and a reaction time of 4 h. The reaction conditions were subsequently adjusted to obtain the best reaction parameters. The prepared catalyst was retrieved and used for catalysing HMF under optimal conditions. After the reaction was completed, the catalyst was separated from the reaction system, thoroughly washed with double-distilled water and oven-dried at 80°C overnight. This experiment was repeated five times.

### 2.5. Analytical methods

The reaction solution may contain various components such as HMF, DFF, HMFCA, FFCA and FDCA. The content of each component can be analysed and tested using high-performance liquid chromatography with an external standard method for quantification. The instrument used was an Aminex HPX-87H chromatographic column. The testing conditions were as follows: room temperature, detection wavelength set at 280 nm, mobile phase consisting of a mixture of acetonitrile and 0.1% acetic acid solution with a volume ratio of 5 : 95. The flow rate of the mobile phase was set to 0.6 ml min^−1^, and the column temperature was maintained at 30°C. The liquid phase column used was Kromasil, C18, 4.60 × 250 mm. Before injection, all test solutions were filtered through a microporous membrane (0.22 µm) in the aqueous phase.

**Figure 1.**
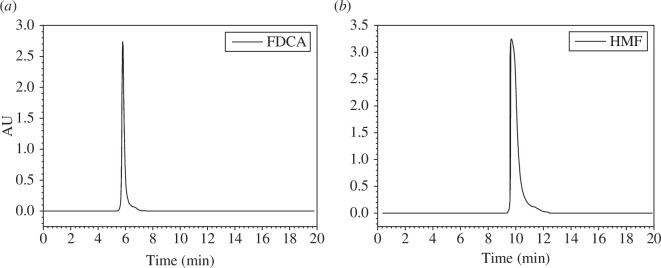
(*a*) High-performance liquid chromatography of FDCA standard liquid. (*b*) High-performance liquid chromatography of HMF standard liquid.

Under these conditions, standard solutions of HMF and FDCA were prepared at a concentration of 0.5 mmol, filtered and then subjected to liquid-phase analysis. The retention times for HMF and FDCA were approximately 10 and 6 min, respectively (figure 1), which were consistent with those reported elsewhere [33].

### 2.6. Methods of calculation


(2.1) $$C_{\text{HMF}}=⟮1-\frac{N_{\text{HMF}}}{N_{0\;\;\text{HMF}}}⟯\times 100\%$$

(2.2) $$Y_{\text{FDCA}}=\frac{N_{\text{FDCA}}}{N_{0\;\text{FDCA}}}\times 100\%$$

(2.3) $$S_{\text{FDCA}}=\frac{Y_{\text{FDCA}}}{C_{\text{HMF}}}\times 100\%$$

*C*_HMF_ represents the conversion rate of HMF, *N*_HMF_ denotes the molar amount of remaining HMF in the reaction solution, *N*_0 HMF_ refers to the molar amount of HMF initially added to the reaction solution, *Y*_FDCA_ represents the yield of FDCA and *S*_FDCA_ stands for the selectivity of FDCA.

## 3. Results and discussion

### 3.1. Morphology

To gain further insight into the surface structure of the modified catalysts, SEM characterization analysis was conducted on the samples to analyse their structures using SEM.

**Figure 2.**
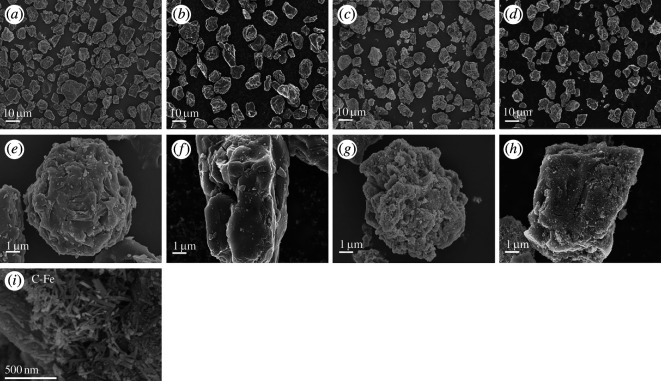
SEM images of catalysts before and after Ru loading (scale = 10 μm): (*a*) activated carbon; (*b*) activated carbon after Ru loading; (*c*) magnetic activated carbon; (*d*) magnetic activated carbon after Ru loading; (*e*) magnified view of activated carbon; (*f*) magnified view of activated carbon after Ru loading; (*g*) magnified view of magnetic activated carbon after Ru loading; (*h*) magnified view of magnetic activated carbon after Ru loading; (*i*) unloaded magnetic activated carbon.

As shown in figure 2*a*, the surface of the unmodified activated carbon is relatively rough, with a large specific surface area. The rough surface and pore structure may be conducive to catalyst loading. No spinel structure was observed on the surface. Similarly, magnetic activated carbon was prepared as illustrated in figure 2*c*. In addition, we have added a 500 nm magnification of figure 2*c*. A comparison indicates that the surface of the magnetic activated carbon is very rough, with a more uniform distribution of magnetic particles. Also, the particles on the surface of magnetic activated carbon are small, basically in the nanometre scale, wrapped around the surface of activated carbon. Additionally, a significant number of rod-like structures are attached to the surface of the magnetic activated carbon, which, based on other reports [34,35], can possibly be iron oxide nanorods. Comparing figure 2*e,f*, it is difficult to determine from the surface observations whether Ru-based metal oxide has been successfully loaded, as the magnetic particles have a similar appearance to the zinc ferrite spinel structure. However, it is noteworthy that the aggregation of surface particles and rod-like structures is significantly reduced in the magnetic activated carbon after Ru loading compared to the non-loaded magnetic activated carbon.

**Figure 3.**
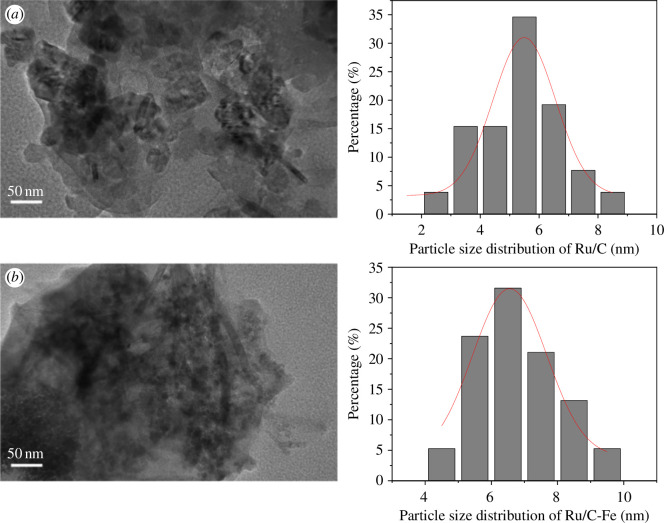
TEM images of Ru loaded on different carbon supports (scale = 50 nm): (*a*) activated carbon after Ru loading; and (*b*) magnetic activated carbon after Ru loading.

The surface structures of the Ru/C and Ru/C-Fe catalysts were characterized using TEM, as depicted in figure 3.

As shown in figure 3*a*, the size of the spinel particles is not clearly discernible, but particle aggregation is evident. The particle shapes are approximately spherical, and the particle size distribution, as shown in the size distribution graph, is concentrated in the 3–7 nm range. Rod-like particles were also found, and after reviewing the literature [35], we can speculate that they are the rod-like oxides on the SEM surface above, which is mutually supportive of the characterization above. As demonstrated in figure 3*b*, the size of the spinel particles is distinctly visible, and a more uniform distribution of particles is observed, indicating an improvement in product dispersion. However, there is still some localized aggregation, consistent with the SEM characterization results, with particle sizes concentrated within the range 5–8 nm. Comparing figure 3*a,b*, it is evident that the loading effect on the activated carbon catalyst is relatively concentrated, while the spinel on the magnetic activated carbon catalyst is uniformly dispersed within the carbon support. The spinel ZnFe_2_O_4_ is paramagnetic, showing a weak magnetic field response, while the magnetic carbon is ferromagnetic. It can be speculated that there might be an interaction between the magnetism of the magnetic carbon and the magnetism of the spinel structure, resulting in the uniform dispersion observed in the loaded magnetic activated carbon.

### 3.2. Phase and elemental analysis

To further explore the loading effect of the carbon supports, EDS analysis was conducted to analyse the elemental composition of the various samples.

**Figure 4.**
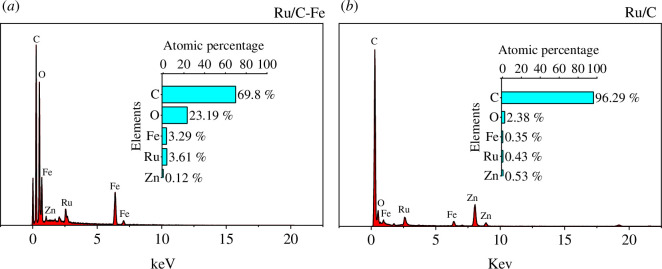
EDS characterization of catalysts: (*a*) Ru/C-Fe; and (*b*) Ru/C.

In line with the aforementioned characterization, it is evident that Ru-based metal oxide was successfully loaded when using both activated carbon and magnetic activated carbon as supports. The Ru proportion in the loaded activated carbon catalyst is 0.43 at%, while the Ru proportion in the loaded magnetic activated carbon is 3.61 at%. It can be hypothesized that the magnetism of the magnetic activated carbon attracts Fe^3+^, leading to an increased loading of the spinel structure on the carbon support, subsequently elevating the Ru proportion. Comparing figure 4*a,b*, it can be observed that the iron loading on the magnetic activated carbon catalyst is higher than that on the activated carbon catalyst, indicating the successful application of magnetic treatment.

**Figure 5.**
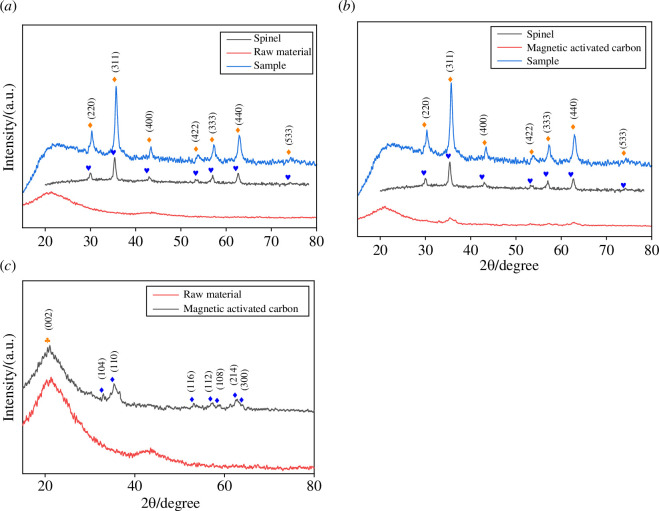
XRD spectra of the samples: (*a*) XRD of spinel, activated carbon and activated carbon catalyst; (*b*) XRD of spinel, activated carbon and magnetic activated carbon catalyst; and (*c*) XRD of activated carbon and magnetic activated carbon.

To investigate the formation of the spinel structure and determine the structure of the carbon supports, XRD was applied to analyse the crystal planes. Figure 5 presents the XRD spectra of the Ru-based catalysts after loading; from these spectra, it is evident that there are strong diffraction peaks at angles 2θ of 30.0°, 35.3°, 42.9°, 53.2°, 56.7°, 62.3° and 74.6°. By comparing these peaks with the Joint Committee on Powder Diffraction Standards (JCPDFS) no. 22-1012 card (it belongs to spinel) [36], they correspond to the crystal planes (220), (311), (400), (422), (333), (440) and (533) of the cubic spinel structure, indicating that the synthesized nanoparticles have a cubic spinel structure. The sharp diffraction peaks without impurity peaks provide strong evidence of the good crystallinity and high purity of the synthesized product [37]. The broadening of the diffraction peaks indicates that the particle size of the sample has reached the nanoscale, demonstrating that the catalyst possesses a stable spinel structure. The broad peak between 20° and 30° corresponds to the (002) crystal plane of the activated carbon, indicating that the generated carbon has an amorphous structure and further confirming the successful loading of the Ru-based metal oxide onto the carbon support. The XRD results for the spinel and activated carbon catalysts show a single spinel structure, indicating the high stability of zinc ferrite. The carbon curve suggests that the introduction of Ru nanoparticles does not alter the nature of the carrier and retains the main characteristic peaks of the zinc ferrite spinel structure. Comparing activated carbon and magnetic activated carbon in figure 5*c* with the JCPDFS no. 33-0664 card, magnetic activated carbon corresponds to the crystal planes (104), (110), (116), (122), (018), (214) and (300) of Fe_2_O_3_, which is consistent with the EDS characterization of the structure, further evincing successful magnetic treatment.

**Figure 6.**
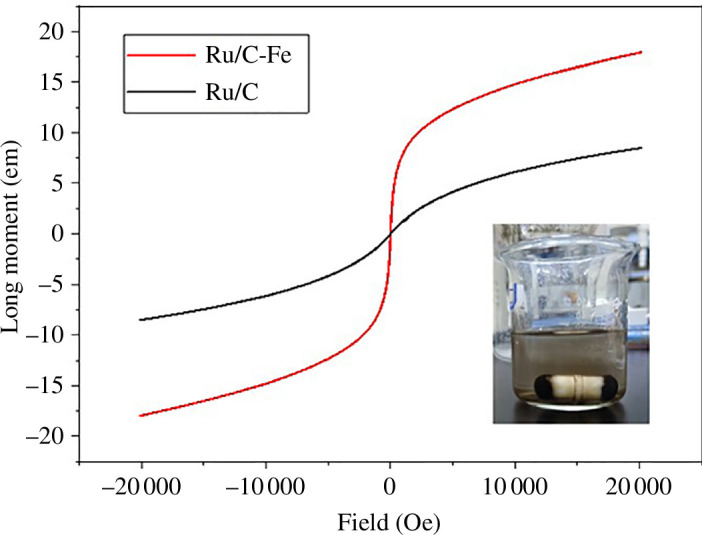
Hysteresis loops of Ru/C catalyst and Ru/C-Fe.

Figure 6 shows the hysteresis loops of magnetic carbon and activated carbon after loading with Ru/C. The results indicate that both materials are soft magnetic materials; the magnetic properties of the magnetic carbon catalyst are stronger than those of the activated carbon catalyst, reaching a saturation magnetization of 17.95 emu g^−1^. However, the magnetic properties of the activated carbon catalyst still reach 8.5 emu g^−1^. This is because the spinel structure has a certain degree of soft magnetism. The results of the hysteresis loops significantly confirm the potential for magnetic separation of Ru/C-Fe.

**Figure 7.**
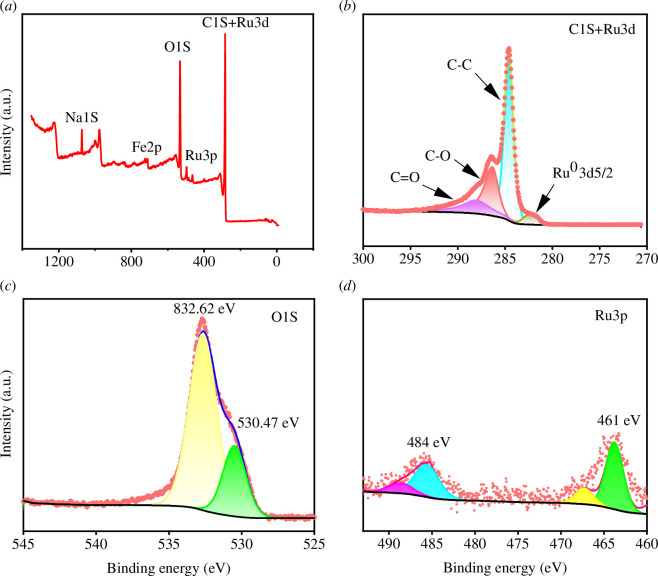
XPS spectra: (*a*) full spectrum of Ru/C; (*b*) high-resolution spectrum of C1s + Ru3d for Ru/C; (*c*) high-resolution spectrum of O1s for Ru/C; and (*d*) high-resolution spectrum of Ru3p for Ru/C.

The binding energies of Fe2p are 713 eV (Fe 2p3/2) and 732 eV (Fe 2p1/2), indicating that the chemical state of Fe in the Ru/C structure is of +3 valence (figure 7). The C1s+Ru3d spectrum can be deconvoluted into three characteristic peaks with binding energies of 284.5, 285.9 and 288.1 eV, corresponding to the C–C bond, C–O bond and C=O bond, respectively. This indicates that the degree of graphitization of the carbon support in the Ru/C catalyst is low, and there are a large number of defect-related or amorphous carbon species present therein [38]. The binding energies of Ru3p are 461 eV (Ru3p3/2) and 484 eV (Ru3p1/2), indicating that the chemical state of Ru is of +3 valence. The O1s curve contains a main peak and a partially overlapping shoulder peak. The lower binding energy O1s peak is located in the range of 530.5–530.9 eV, and the higher binding energy O1s peak is located in the range of 532.6–534.1 eV. The O1s peak at 532.6–534.1 eV is attributed to ZnFe_2_O_4_-Ru [39].

### 3.3. Influences of experimental conditions on catalytic performance

Before exploring the following conditions, as described in the previous text, the initial experimental conditions were set as follows: HMF at 0.5 mmol, the catalyst dosage of 50 mg, the chosen solvent was DMSO (3 ml) and the reaction temperature was set to 110℃, with a reaction time of 4 h.

**Figure 8.**
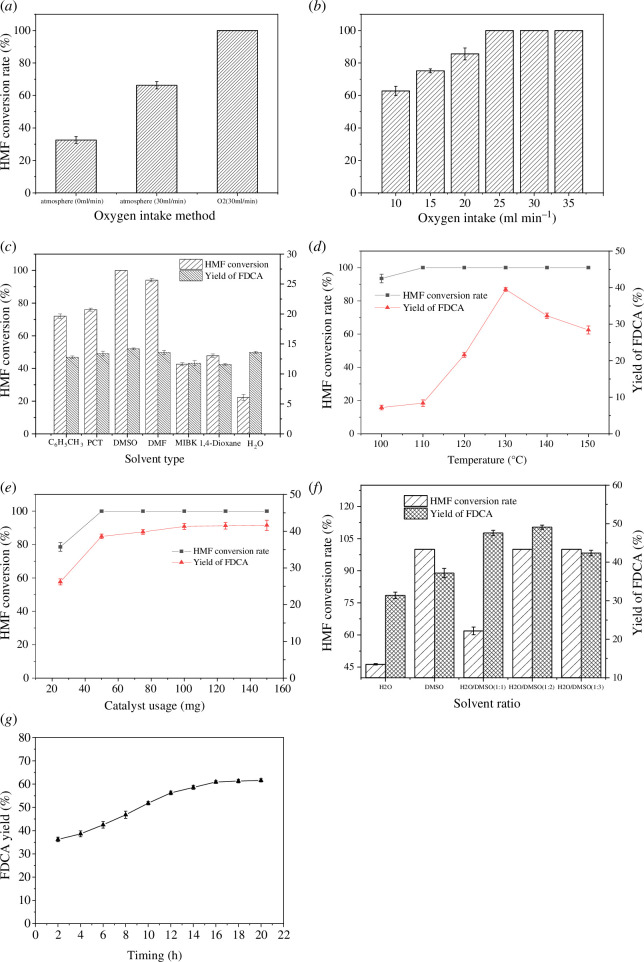
(*a*) Effect of the method of oxygen introduction on HMF conversion; (*b*) effect of the oxygen flow rate on HMF conversion; (*c*) effect of solvent selection on the catalytic performance of Ru/C; (*d*) effect of temperature on the catalytic; (*e*) effect of Ru/C dosage on HMF conversion; (*f*) effect of different solvent ratios on HMF conversion; and (*g*) effect of reaction time on FDCA yield.

The oxidation of HMF to FDCA is an oxidative reaction, and the oxidant used is oxygen. First, the introduction method of oxygen was investigated. Three sets of experiments were conducted: an open system, continuous air introduction and continuous oxygen introduction. By comparing the results, the effects of different methods of oxygen introduction on HMF conversion were observed. As shown in figure 8*a*, continuous oxygen introduction is the optimal method.

After identifying the optimal method of oxygen introduction, the amount of oxygen introduced was studied. Oxygen flow rates were set from 10 to 35 ml min^−1^ in increments of 5 ml min^−1^. Figure 8*b* shows that after the oxygen flow rate reaches 25 ml min^−1^, the HMF conversion rate remains relatively constant. This finding indicates that the optimal oxygen flow rate is 25 ml min^−1^.

According to reports, solvents play a crucial role in chemical reactions owing to their various properties such as polarity, dielectric constant, steric hindrance and acidity–basicity. The selection of solvent is particularly important for organic reactions. In this study, to explore the optimal conditions for catalyst-catalysed reactions, several common and different types of organic solvents were selected to assess the effects of solvents on the catalytic ability of the catalyst. As shown in figure 8*c*, when the solvent is DMSO, both the conversion rate and the yield of the catalyst are higher than those in other solvents. Therefore, DMSO is deemed to have the optimal solvent. However, when the solvent is water, although the conversion rate of HMF is low, the yield is very high. It is speculated that water molecules facilitate the oxidative reaction. Further experiments will be conducted with different ratios of water to explore such effects.

Temperature is crucial to this reaction. As illustrated in figure 8*d*, a temperature range of 100–150°C was established in increments of 10°C to investigate the optimal reaction temperature. As the temperature was increased from 100 to 130°C, there was a slight increase in the conversion rate, which then remained stable. The yield of FDCA increased as the temperature rose, reaching a peak at 130°C and then started to decrease. This indicates that the optimal temperature was 130°C.

Next, the optimal catalyst dosage was explored. A mass range of 20–160 mg was adopted (figure 8*e*); when the catalyst dosage increased to 100 mg, it reached its maximum, and further increases in the catalyst dosage had little effect on the yield.

Considering the previous finding that the use of water as a solvent resulted in a high yield of FDCA, despite a low conversion rate, different ratios of DMSO to H_2_O were used as solvents to further investigate the effect on the FDCA yield. The results, as shown in figure 8*f*, indicate that the FDCA yield was highest when the solvent was H_2_O/DMSO (1 : 2).

Finally, the influence of reaction time on the oxidative reaction was studied. As illustrated in figure 8*g*, a time range of 0–22 h was adopted. As the reaction time was extended from 4 to 16 h, the yield of FDCA gradually increased. When the reaction time reached 16 h, the FDCA yield was the highest, reaching 40.9%. Further increasing the time did not significantly increase the FDCA yield.

The experimental results above suggest that the optimal conditions for the synthesis of FDCA through the oxidation of HMF are as follows: 0.5 mmol (63 mg) of HMF, in an environment at 130°C, with a solvent consisting of H_2_O/DMSO (1 : 2) with a volume of 3 ml, a catalyst dosage of 100 mg, an oxygen flow rate of 25 ml min^−1^ and a reaction time of 16 h.

### 3.4. Influence of reusability on catalytic performance

**Figure 9.**
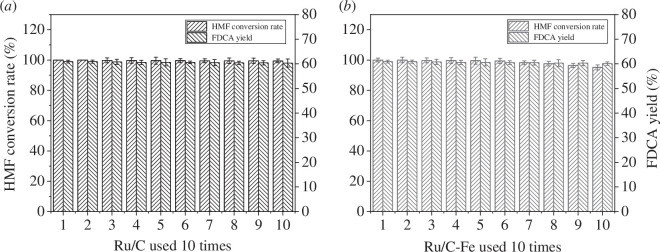
Effect of reusability on the catalytic performance: (*a*) Ru/C reused 10 times; and (*b*) Ru/C-Fe reused 10 times.

The reusability of catalysts is an important indicator to estimate their stability. In this study, the catalytic performance of Ru/C and Ru/C-Fe in the repeated use of HMF was evaluated over 10 cycles (figure 9). The results indicate that after 10 cycles of reuse, both Ru/C and Ru/C-Fe show a slight decrease in the HMF conversion rate and FDCA yield, with the FDCA yield of Ru/C-Fe catalyst decreasing from 60.9% to 60.1%. These experiments indicate that Ru/C-Fe possesses remarkable stability.

### 3.5. Comparison

Comparison of our products with Yang *et al*.’s [40] products is represented by the following graphs.

**Figure 10.**
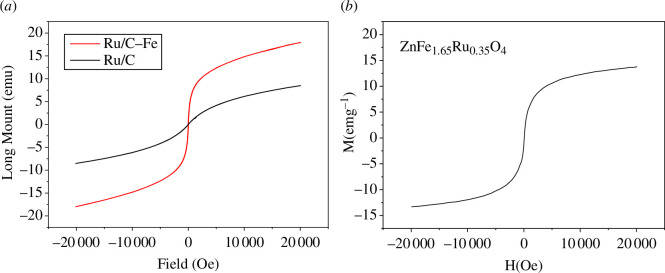
Comparison of magnetic properties. (*a*) Magnetic properties of Ru/C and Ru/C-Fe; (*b*) Magnetic properties of ZnFe_1.65_Ru_0.35_O_4_.

From the comparison of figure 10*a,b*, we can see that the magnetic properties of Ru/C are lower than that of ZnFe_1.65_Ru_0.35_O_4_, but the magnetic properties of Ru/C-Fe are higher than that of Ru/C. This indicates that the magnetic separation ability of Ru/C-Fe is stronger than that of ZnFe_1.65_Ru_0.35_O_4_.

**Figure 11.**
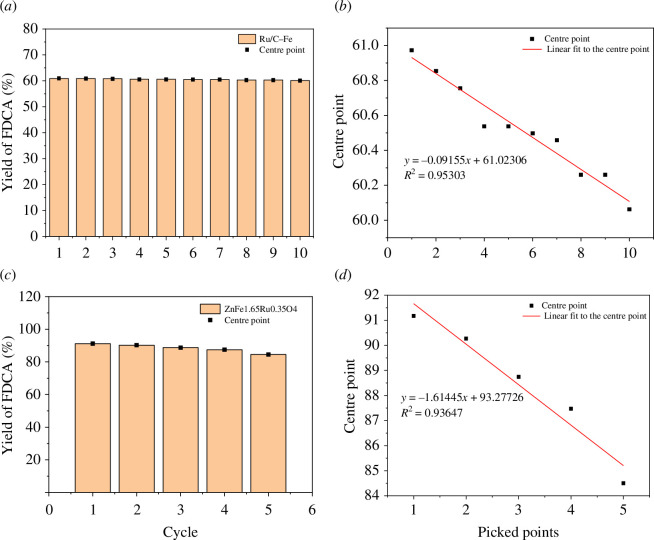
Comparison of catalytic efficiency and reusability. (*a*) Ru/C-Fe repeated use times; (*b*) Ru/C-Fe catalytic efficiency; (*c*) ZnFe_1.65_Ru_0.35_O_4_ repeated use times; (*d*) ZnFe_1.65_Ru_0.35_O_4_ catalytic efficiency.

Comparing figure 11*a-d*, we can observe that the catalytic efficiency of ZnFe_1.65_Ru_0.35_O_4_ is higher than that of Ru/C-Fe. We speculate that it may be the influence of SO_3_H, as SO_3_H can provide more active sites, which is beneficial for improving catalyst efficiency. However, at the same time, by comparing the trend line equations, we can see that the catalytic performance of Ru/C-Fe decreases less than that of ZnFe_1.65_Ru_0.35_O_4_, which means that the reusability of Ru/C-Fe is higher than that of ZnFe_1.65_Ru_0.35_O_4_.

Comparing with the magnetic Ru-based metal oxide catalyst prepared by Yang, this article loaded the spinel structure on the carbon carrier on the basis of it, and the yield of FDCA was reduced to a certain extent in terms of catalytic efficiency. However, the ZnFe_1.67_Ru_0.3_O_4_ nanoparticles are easy to agglomerate and not easily dispersed owing to their large specific surface energy, and the ferrate is easy to corrode and precipitate metal ions in acid with strong acidity (pH = 2–3), resulting in the loss of Ru in the active site, which leads to a decrease in catalytic performance. The use of carbon carriers for loading is not only inexpensive and easy to recover the active components but also conducive to overcoming the shortcomings of its strong acid conditions, which can solve the deficiencies of the spinel structure. Also, the product has higher stability and reuse ability.

In conclusion, although the catalytic efficiency of Ru/C-Fe is weaker than that of ZnFe_1.65_Ru_0.35_O_4_, its reusability and stability are stronger than that of ZnFe_1.6 5_Ru_0.35_O_4_. Ru is a precious metal, and the ability to be reused many times and to maintain its stability also improves the utilization efficiency of Ru.

## 4. Conclusions

A mixture catalyst of spinel and Ru-based metal oxide nanoparticles was successfully synthesized by alkaline co-precipitation. The Ru-based catalysts with excellent reactivity, stability and strong magnetism were obtained by loading them onto activated carbon and magnetic activated carbon carriers. The particle size was concentrated in the range of 5–8 nm, with a loading content of 3.61 at%, exhibiting strong soft magnetism. This catalyst proved to be highly effective in the catalytic conversion of HMF to FDCA. The optimal reaction conditions were determined as follows: a catalyst dosage of 100 mg, a solvent mixture of H_2_O/DMSO (1 : 2), a reaction temperature of 130°C, an oxygen flow rate of 25 ml min^−1^ and a reaction time of 16 h, with a maximum yield of 60.9%. The product yield remained stable over 10 use/reuse cycles.

Thus, the catalyst prepared in this study addresses the stability issues of some Ru-based catalysts in the HMF oxidation to FDCA reaction, while also enhancing the reusability of the catalyst. This carbon-supported spinel-type catalyst exhibits not only remarkable stability but also magnetic properties, facilitating easy separation and high reusability. It can be applied not only to Ru-based catalysts but also to the preparation of other noble metal catalysts.

## Data Availability

The data presented in this study, together with relevant code for this research work are stored in Dryad [41].
